# ORGANOTYPIC BRAIN SLICE CULTURES: A REVIEW

**DOI:** 10.1016/j.neuroscience.2015.07.086

**Published:** 2015-08-05

**Authors:** C. HUMPEL

**Affiliations:** Laboratory of Psychiatry and Experimental Alzheimer’s Research, Department of Psychiatry and Psychotherapy, Medical University of Innsbruck, Anichstrasse 35, A-6020 Innsbruck, Austria

**Keywords:** organotypic, whole-brain cultures, dopaminergic, cholinergic, vascular

## Abstract

*In vitro* cell cultures are an important tool for obtaining insights into cellular processes in an isolated system and a supplement to *in vivo* animal experiments. While primary dissociated cultures permit a single homogeneous cell population to be studied, there is a clear need to explore the function of brain cells in a three-dimensional system where the main architecture of the cells is preserved. Thus, organotypic brain slice cultures have proven to be very useful in investigating cellular and molecular processes of the brain *in vitro*. This review summarizes (1) the historical development of organotypic brain slices focusing on the membrane technology, (2) methodological aspects regarding culturing procedures, age of donors or media, (3) whether the cholinergic neurons serve as a model of neurodegeneration in Alzheimer’s disease, (4) or the nigrostriatal dopaminergic neurons as a model of Parkinson’s disease and (5) how the vascular network can be studied, especially with regard to a synthetic blood–brain barrier. This review will also highlight some limits of the model and give an outlook on future applications.

## INTRODUCTION

*In vitro* cell cultures are an important technique for studying large quantities of homogeneous cells in an isolated environment. Thus, the *in vitro* culturing of primary dissociated neurons, astrocytes or oligodendrocytes, or endothelial cells has become an essential method employed by many neuroscientists. Especially also with a view to the increasing number of animal research experiments, *in vitro* cultures permit the number of experiment animals and their suffering to be markedly reduced. Primary *in vitro* cell cultures allow survival, morphology, function as well as the influence of toxic or protective chemicals to be studied. However, isolated cells do not reflect the nature of the organism due to the isolation and lack of contact with other cells. Thus, over the last decades organotypic cultures have been found to be an important step forward in simulating more *in vivo*-like situations. Organotypic cultures allow several aspects of structural and synaptic organization of the original tissue to be preserved. This review will summarize historical and methodological aspects of organotypic cultures and discuss whether cultures containing dopaminergic or cholinergic neurons can serve as *in vitro* models of Parkinson’s or Alzheimer’s disease, respectively.

The term “organotypic” was first published in 1954 in a report on differentiation of the chick embryo eye (Reinbold, 1954), followed by a report on the lung and heart (Loffredo and Sampaolo, 1956) and the intestine (Monesi, 1960). The first description of CNS tissue focused on rat hypophysis (Bousquet and Meunier, 1962) and was followed by the pioneering work of Crain (1966 and 1972) on the development of “organotypic” bioelectric activities in CNS tissues during maturation. Interestingly, the first detailed description of brain tissue was published using organotypic cerebellum (Wolf, 1970; Hauw et al., 1972).

A first technical description was given by Boyd (1971) in a report on a chamber for organotypic cultures used to grow large volumes of tissue. This chamber was further modified and optimized as a tissue plate (Ansevin and Lipps, 1973). The breakthrough was made by Gähwiler’s group, who cultured organotypic brain slices using the **roller tube technique** (Gähwiler, 1981a,b; Gähwiler, 1988; Braschler et al., 1989; Gähwiler et al., 1997, 2001; Victorov et al., 2001). The method was modified and optimized by Stoppini et al. (1991), who found that organotypic brain slices survive well when cultured on **semipermeable membranes.** Meanwhile, this method has been used and adapted by several research groups including ours (Bergold and Casaccia-Bonnefil, 1997; Noraberg, 2004; De Simoni and Yu, 2006; Marx, 2010; Ullrich et al., 2011; Ullrich and Humpel, 2011; Shamir and Ewald, 2014). As an attractive alternative the **in oculo model** was developed, which allows three-dimensional tissue grafts in the anterior eye chamber to be studied.

### (a) Organotypic tissue slices in the anterior eye chamber

The anterior chamber of the eye is an easily accessible site, and it has been well documented that grafting of brain tissue into the lateral angle between the cornea and the iris provides a perfect environment for survival and growth. This in oculo model (Hoffer et al., 1974; Olson et al., 1985) allows various brain tissues (e.g. the hippocampus, cerebellum, locus coeruleus, substantia nigra, cortex) to be studied in total isolation. The anterior surface of the rodent iris is highly vascularized, which supports survival of transplanted brain tissues. This model allows tissue growth, trophic effects and interactions of different brain areas to be studied. Indeed, models of neuronal pathways have been constructed, such as e.g. the nigrostriatal dopamine, coeruleospinal or the cholinergic septohippocampal pathways. Thus, this in oculo model allows isolated brain tissues to be investigated *in vivo* directly in the rodent eye. The tissue can be directly followed by simple stereomicroscope observation, each animal can be given grafts in both eyes, vision is not disturbed and the whole procedure is rapid and simple so that a large number of animals can be generated. The major disadvantage of this model is, however, that it is still a severe animal experiment and does not reduce the number of animal experiments. Moreover, in some situations the nerve fibers innervating the iris hamper or stimulate the in oculo grafts.

### (b) Roller tube technique

Initially, organotypic brain slice cultures were established using the roller tube technique. The brain slices are placed on coverslips in a drop of plasma to which thrombin is added to make the plasma coagulate, and thereby “glue” the brain slice to the coverslip. With proper use of plasma and thrombin very few slices are lost by falling off, – yet they may still to some extent disorganize, die and disappear.

### (c) Semipermeable membrane technique

The semipermeable membrane technique is a modification of the roller tube technique. In contrast to the roller tube technique, slices are placed on a semipermeable membrane and medium is added below the membrane. Lack of or delayed attachment and falling off is not a problem for brain slices grown by this technique, given that the inserts with the semipermeable membrane are kept in regular incubators, and only moved and handled at medium change. The membrane technology has the big advantage that it employs two compartments separated by a permeable membrane. Cells can be cultured in the lower compartment and slices cultured on the upper membrane. The size of the pores in the membrane determines which substrates/cells can diffuse into the slice or whether slices can be directly co-cultured with other cells, e.g. forming a blood–brain barrier (BBB) (see below). Usually, slices are never fully soaked in medium but are covered with only a small film of medium at the upper surface.

## METHODOLOGICAL ASPECTS USING THE MEMBRANE TECHNIQUE

**Fig. 1:**
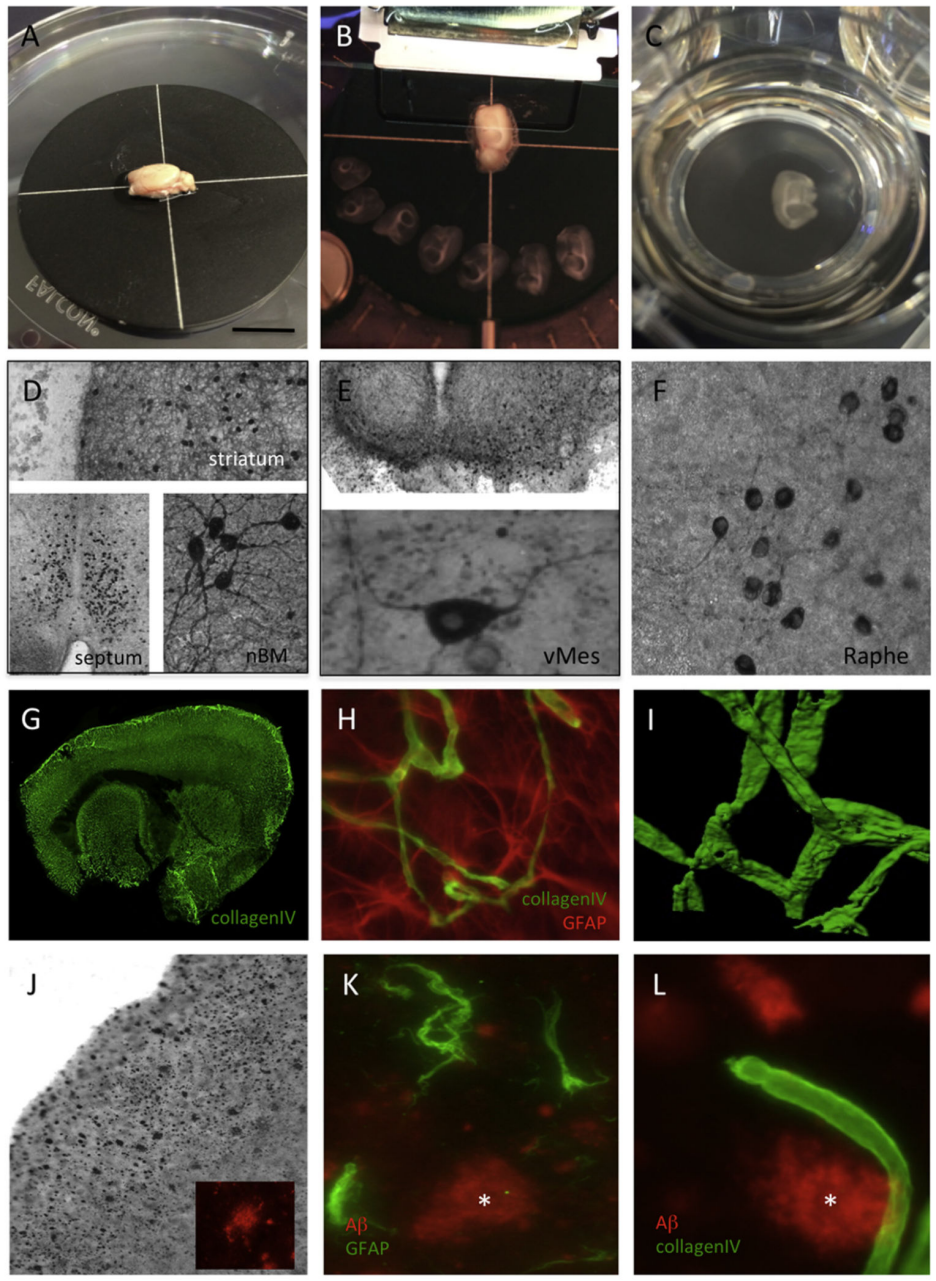
Organotypic brain slices are prepared from whole postnatal or adult brains (A), and 100- to 400-μm-thick sections are cut with a vibratome (B) and placed in an insert with 0.4-μm semipermeable pores (C). Cholinergic neurons stained for choline acetyltransferase^+^ neurons were found in the striatum, septum and basal nucleus of Meynert after incubation with 10 ng/ml nerve growth factor (NGF) for two weeks (D). Dopaminergic tyrosine hydroxylase^+^ neurons survive well in the ventral mesencephalon (vMES) when incubated with 10 ng/ml glial cell line-derived neurotrophic factor (GDNF) for two weeks (E). Immunostainings for tryptophane hydroxylase show raphe neurons after two weeks in culture (F). Brain slices display a strong vascular network, as seen by collagen IV staining (Alexa-488, green) (G). The vascular system is in direct interaction with astrocytes co-stained for collagen IV (Alexa-488, green) and glial fibrillary acidic protein (GFAP, Alexa-546, red) (H). High-power confocal microscopy shows a collagen IV^+^ (Alexa-488, green) vessel in the organotypic slices after two weeks in culture (I). Adult whole-brain organotypic slices (110-μm thick) of the APP_SweDI Alzheimer mouse model were cultured for two weeks, stained for beta-amyloid using Alexa-546 (red) (J, K, L) and co-stained with GFAP (Alexa-488, green) for reactive astrocytes (K) or collagen IV using Alexa-488 (green) (L). Note that sections in Figure D-F&J were stained using the chromogenic substance DAB, while the sections in G–L underwent fluorescent staining. Scale bar in A = 980 μm (A–C), 300 μm (D-striatum), 600 μm (D-septum), 90 μm (D-nBM); 600 μm (E upper), 20 μm (E lower), 70 μm (F), 250 μm (G), 60 μm (H), 30 μm (I), 240 μm (J), 36 μm (K) and 24 μm (L).

A short technical description is given in the following section focusing on the membrane technology (Fig. 1). The animals (e.g. postnatal P5-P10) are rapidly sacrificed, the head briefly placed in 70% ethanol and the brains dissected. The brains are glued (e.g. Glue Loctite) to the chuck of a water-cooled vibratome (e.g. Leica VT1000A) and trimmed close with a commercial shave razor. Under aseptic conditions, 100- to 400-μm-thick whole-brain (sagittal or coronal) sections are cut and collected in sterile medium. The organotypic slices are carefully placed in a 0.4-μm membrane insert (Millipore PICM03050) in a 6-well plate. Optional slices can also be first placed on a sterile 0.4-μm pore extramembrane (Millipore HTTP02500). Brain slices (1–3 per well depending on size) are cultured in 6-well plates (Greiner) at 37 °C and 5% CO_2_ and are incubated for minimum two weeks with the medium changed once or twice per week. Slices are usually cultured with or without growth factors to support survival of specific neurons. At the end of the experiment, slices are fixed for 3 h at 4 °C in 4% paraformaldehyde (PAF)/10 mM phosphate-buffered saline (PBS) and then stored in PBS/sodium azide at 4 °C until use. Alternatively, brain slices can also be cut into 200- to 400-μm-thick sections using a Mac Illwain tissue chopper, with six to eight slices cultured on the membrane.

### Medium to culture organotypic brain slices

We usually add 1.2 ml/well of the well-established culture medium according to Stoppini et al. (1991): 50% MEM/HEPES (Gibco), 25% heat-inactivated horse serum (Gibco/Lifetech, Austria), 25% Hanks’ solution (Gibco), 2 mM NaHCO_3_ (Merck, Austria), 6.5 mg/ml glucose (Merck, Germany), 2 mM glutamine (Merck, Germany), pH 7.2. Horse serum has a positive influence on tissue flattening, providing positive survival promoting effects on neurons, astroglia or microglia in organotypic brain slices. However, in some cases the medium must be modified (Kim et al., 2013). Initially, Annis et al. (1990) reported on a chemically defined medium for organotypic slice cultures and often there is a need to further optimize or adapt the medium for specific conditions, e.g. when using glucose–oxygen deprivation or serum-deprivation or when culturing slices from adult donors.

### Age of donors for organotypic slice cultures

Donor age is very important for organotypic slice cultures. It is well known and established that tissue or cells from embryonic donors survive better and also increase in size.

#### (a) Embryonic donors >E14

Using in oculo transplants Henschen et al. (1985) showed that E14 tissue increases to eightfold its initial size, while E16 increases to threefold its initial size and E17 increases to twice its initial size. There are clear indications that primary dissociated neurons are well established from embryonic donors and survive well. While brain slices from embryonic donors also survive well on membrane inserts, usually organotypic brain slices are derived from postnatal donors due to their higher maturity.

#### (b) Postnatal donors (<P12)

For organotypic brain cultures postnatal day 10–12 donors are recommended because of better morphology, increased survival and more stable/homogeneous susceptibility in lesion models. Plenz and Kitai (1996) developed cortex striatum mesencephalon (triple) organotypic cultures from rat postnatal day 0–2 brain and modified the “roller tube technique” by embedding slices in a plasma/thrombin clot on a Millicell membrane on a cover slip. Organotypic slice cultures from the mesencephalon, striatum, hippocampus and cerebellum were prepared from late fetal (E21) to P7 rats and cultured for three to 60 days using the roller tube technique (Ostergaard et al., 1990). In our hands, we noticed that P8–P10 brains provide a perfect time window for establishing brain slice cultures on membranes that survive well, even for several months (Marksteiner and Humpel, 2008).

#### (c) Adult donors

In my opinion, there is a clear need to culture brain tissue from adult donors. Unfortunately, not many papers have been published on intact functional adult organotypic slices. Most authors who claim to successfully use adult slices investigate mainly processes in acute very short-living adult slices (Lossi et al., 2009). At any rate, for long-term cultures the culture conditions need to be optimized for adult organotypic slices. Kim et al. (2013) claimed to culture adult hippocampal slices in serum-free medium. Wilhelmi et al. (2002) used a CSF-like medium and reported good culturing of adult hippocampal tissue for at least six days. We ourselves have good experience in culturing slices from adult mice. However, one needs to be very careful to culture thin (approx. 100–120 μm) sections. Using such 110-μm-thin adult sections from transgenic Alzheimer mice we were able to show that beta-amyloid plaques are still evident and surrounded by reactive astrocytes and microglia (Humpel, 2015). However, we were not able to prolong the survival of sensitive neurons (such as e.g. dopaminergic or cholinergic neurons), even when incubated with growth factors. Indeed, there is a clear need to develop and characterize adult organotypic brain sections, either for the purpose of studying slices from transgenic animals (Duff et al., 2002; Quadros et al., 2003; Mewes et al., 2012) or, more importantly, slices from human postmortem or biopsy brains (Eugene et al., 2014).

### Acute versus long-term cultures

When performing experiments with brain slices the question arises: when to do the analysis? In general, slices can be studied immediately after dissection (acute) or after having been grown for longer times to chronological adult age and maturation. The analysis of acute slice experiments (not culturing) has the advantage of providing insights into cellular or molecular processes of rapidly sacrificed animals and may display a near *in vivo* situation. For these experiments, slices must not be cultured, but endogenously released (toxic) molecules are normally washed out before the experiment starts. Usually, electrophysiology or release experiments can be performed or experiments after short incubation with stimuli, where slices are then extracted by e.g. sonication or lysis and then further analyzed. For this purpose slice thickness and survival are not relevant. Moreover, slices from adult donors or even postmortem tissue can be processed. However, in the case of organotypic brain slice cultures these slices need to be cultured for at least ten to 14 days to guarantee that they are not activated by endogenous release of e.g. calcium or glutamate and that reactive astrogliosis is minimized. Further, developing slices need time for maturation and stabilization of intrinsic axonal projections. Only such “resting non-activated” brain slices are useful for further investigation.

### Flattening as a means of macroscopic survival

The organotypic sections attach to the membranes a few days after being transferred to the membrane inserts and are fully attached to the membrane after two weeks *in vitro*. This is important because the slices flatten and become transparent, which is an important macroscopic sign that the slices are healthy. However, using the lack of flattening alone and measuring the thickness of the cultured slices as a criterion for lack of slice culture survival after set-up appears complicated. More importantly, the general change in color and transparency from whitish-opaque at the time of set-up to a transparent gray during the first week is an important criterion for evaluating whether the slices are well-cultured. Non-surviving cultures or parts that do not survive remain whitish-opaque. Furthermore, outgrowth of cells from the edge of the living slices is another important criterion for evaluating good slices. Thick and not flattened slices should normally be withdrawn from the experiment. In our hands we observed a time window of postnatal 8–12 days, during which slices flatten down very well. The differences observed in “flattening out” of the brain slice cultures of different donor age can be explained by developmental stage differences in growth ability and texture of the slices. However, we recently (Humpel, 2015) started to culture slices from adult animals and sectioned 110-μm-thick slices, some of which display good functional activity. Anyone who wants to measure tissue slice thickness can consult the report by Guy et al. (2011).

### Survival of cells in slices

The survival of cells in the organotypic slice cultures is the most important criterion to consider. In general, the older the animal, the less tissue survives and the greater the cell death is. While this is not the principal problem for astrocytes or endothelial cells, neuronal survival is the major challenge. Several parameters influence the survival of neurons, such as tissue age, medium composition including growth factors and serum, thinning of the tissue slice, preparation speed, sterility, health of the donor animals, etc. The lack of thinning is the most important first macroscopic criterion of cell death or necrosis. However, to get more information on cellular viability, tissue slices must be counterstained with cell death-specific agents. Several fluorescent dyes are commercially available to directly study the viability of cells in living slices under the inverse fluorescence microscope. The most frequently used dyes are propidiumiodine, ethidiumbromide, SYTOX dyes, Hoechst dyes, acridinorange or DAPI or annexin V (see for more details Lossi et al., 2009). The advantage of these “live cell stainings” is that the slices can be investigated directly under the microscope and can be further cultured. However, all these dyes are not specific for a particular cell type and do not give information on neuronal survival. In order to study cell-specific death or apoptosis, slices need to be fixed (usually 3 h 4% PAF) and then counterstained for cell-specific markers (e.g. microtubuli associated protein-2 for neurons, glial fibrillary acidic protein for astrocytes or CD11b for microglia or laminin for vessels). In some cases it is very useful to investigate apoptotic cell death. Several different specific apoptotic markers are available, such as e.g. cleaved caspases or PARP-1, FADD, proto-oncogenes or mitochondrial enzymes (see for details Lossi et al., 2009). There are several examples of published papers investigating apoptotic cell death in organotypic brain slices, such as e.g. after stimulation with phencyclidine (Timpe et al., 2014), microRNAs (Irmady et al., 2014), berberine (Simões Pires et al., 2014), manganese (Xu et al., 2014) or iron (Dixon et al., 2012), tunicamycin (Leggett et al., 2012), palmitoylethanolamide (Scuderi et al., 2012), cathepsins (Ceccariglia et al., 2011), prostaglandins (Koch et al., 2010) or PARP-2 inhibitors (Moroni et al., 2009). Further, a nice work shows that in the organotypic postnatal mouse cerebellar cortex the anti-apoptotic protein BCL-2 is regulated by autophagy modulating neuronal survival (Lossi et al., 2010). Thus, many papers have been published on exploring necrosis or apoptosis in organotypic brain slices, however, cannot be completely reviewed here without being complete.

### Applications using organotypic brain slices

Organotypic brain slice cultures offer many possibilities to study many types of brain cells *in vitro*. This review will highlight only a few possibilities from the many publications showing the strong potency of these *in vitro* cultures. Several applications have been reported, such as e.g. repeated multi-electrophysiological recordings and stimulations (Egert et al., 1998; Jahnsen et al., 1999; Karpiak and Plenz, 2002; Dong and Buonomano, 2005), or gene transfer techniques (Ridoux et al., 1995; Thomas et al., 1998; Murphy and Messer, 2001), retrograde tracing using fluorescent dyes (Ullrich and Humpel, 2011), or long-term live imaging (Gogolla et al., 2006). Organotypic brain slices can be analyzed using all common neurobiological methods. Slices can be detached from the membranes and extracted (e.g. by sonification or lysis) for use for ELISAs, RT-PCR, HPLC etc. PAF-fixed slices are easy to handle and free-floating; they can be immunohistochemically stained (chromogenic or fluorescent), transferred to glass slides and cover slipped or directly analyzed under an inverted microscope (Ullrich et al., 2011). Slices can also be analyzed by in situ hybridization, although this is a bit tricky because sometimes only fresh (unfixed) slices can be used (Gerfin-Moser and Monyer, 2002; Ullrich et al., 2011).

In our research group we usually conduct neuroprotection and neurotoxicity assays. Organotypic brain slices can be easily used to test neuroprotective molecules such as e.g. growth factors or neuroactive drugs (Sundstrom et al., 2005; Drexler et al., 2010). Usually, brain slices are incubated from the beginning of culturing with the respective neuroprotective drugs for e.g. two weeks and then analyzed. Organotypic brain slices are also well-established models for neurotoxological screenings (Noraberg, 2004). In such an experiment slices are cultured for at least two weeks under optimal conditions (if necessary with growth factors) to guarantee a stable well-established non-inflamed and non-reactive model. Then we usually withdraw the growth factor for three days and subsequently add an exogenous degenerative toxic stimulus before incubating for three to 14 days. We have observed that organotypic brain slices need markedly higher doses of a toxic stimulus than do primary cells.

## AXOTOMY, LOSS OF TARGET AND SYNAPTOGENESIS AND THE HIPPOCAMPUS

Several brain areas have been cultured as organotypic brain slices: cortex (Giesing et al., 1975), striatum (Ostergaard et al., 1995), substantia nigra (Whetsell et al., 1981; Kida, 1986), raphe (Jonakait et al., 1988; Hochstrasser et al., 2011), locus coeruleus (Knöpfel et al., 1989), the basal forebrain (Robertson et al., 1997) and the suprachiasmatic nucleus (Wray et al., 1993) as well as several others, such as the hypothalamus, thalamus, supraoptic nucleus or olfactory system.

The most explored brain area in organotypic cultures is the hippocampus. In 1973, LaVail and Wolf (1973) reported for the first time on the postnatal development of mouse dentate gyrus. Several research groups have explored the morphology, histogenesis and ultrastructure as well as the functional role of the hippocampal formation (Zhabotinski et al., 1979; Gähwiler, 1981a; Beach et al., 1982; Gähwiler and Hefti, 1984; Zimmer and Gähwiler, 1987; del Rio et al., 1991; Buchs et al., 1993; Muller et al. 1993): the hippocampal organotypic formation served as a model for studying neurodegeneration (oxygen glucose deprivation, oxidative stress, posttrauma, anoxia, asphyxia, hypothermia, hypoglycemia, ischemia, epileptogenics, ethanol), neurotoxicity (N-methyl-d-aspartate (NMDA) toxicity, metals), infections, as well as neuroinflammation and neuroprotection. Furthermore, spine morphology, dendritic growth, mossy fiber sprouting and synaptic plasticity including long-term potentiation, neurogenesis and stem cells have been explored in the organotypic hippocampus.

Although in organotypic brain slice cultures the cells maintain their connections, they lose their target innervation because the slices are an axotomized system. This axotomy is the major disadvantage of the slice culture system, because axotomy causes neuronal cell death. Especially embryonic or neonatal brains are very sensitive for axotomy, because they are dependent on their targets and the supply of target-derived neurotrophic factors. In mature brains axotomy may lead to regenerative responses without any severe neuronal death, due to local production and secretion of growth factors. Clearly some of the neurons in the cut and cultured slices maintain their axonal connections to other neurons within the given tissue slice, just as they and other neurons loose normal afferent connections from more distant areas and levels not included in the slice. Loss of afferent connections to neurons within the cultured slices, combined with the loss of efferent connectivity to normal (outside) target areas, elicits a reorganization and expansion of intrinsic axons to “denervated” intrinsic terminal fields. Definitely, the addition of exogenous growth factors is recommended for specific subsets of neurons, such as e.g. nerve growth factor (NGF) for cholinergic neurons (see below). The need for growth factor supplements for a specific tissue and neuronal population needs to be determined experimentally, possibly also a combination of growth factors. We also experienced that some neuronal populations, e.g. serotonergic neurons, also survive without growth factor addition. However, not all neurons in a brain slice are axotomized, e.g. cholinergic interneurons in the striatum can be studied as an isolated non-axotomized system. These neurons will lose synaptic innervations from e.g. cortex or mesencephalon and be functionally dysregulated.

However, on the other hand, axotomy also allows reactive synaptogenesis and neuronal sprouting in organotypic brain slice cultures to be studied, such as e.g. mossy fiber reorganization in hippocampal slice cultures (Zimmer and Gähwiler, 1984; Gähwiler, 1981a). In fact, the hippocampus is a brain region of specific interest for the study of synapse formation, especially mossy fiber sprouting. The pioneering work of Stoppini et al. (1993) showed neurite outgrowth and reactive synaptogenesis in one- to three-week old hippocampal organotypic cultures. They (Stoppini et al., 1993) observed a thin scar within six days of lesion formation, the presence of numerous degenerative and regenerative processes after one day and many new functional synaptic contacts and complete recovery of transmission within three to six days. These data were extended by Robain et al. (1994), who showed that mossy fibers expanded their terminal fields and invaded the CA3 region and dentate gyrus. Muller et al. (1994) found that the sprouting reaction was triggered by the expression of neuronal cell adhesion molecules, playing an important role in neuronal sprouting and synapse regeneration. Such an axotomy slice model also allows new innervations to be studied in co-culture models. del Río et al. (1996) found that Cajal-Retzius cells survive in long-term single hippocampal cultures, but that fewer cells survive when coupled to the entorhinal cortex, more likely simulating an *in vivo* situation. Taken together, all these experiments nicely show that long-term organotypic slice cultures are an attractive potent model for studying reactive synaptogenesis and neuronal plasticity, cellular atrophy and age-related processes (Bahr, 1995).

## NEURODEGENERATION OF CHOLINERGIC NEURONS AS A MODEL FOR ALZHEIMER’S DISEASE?

Cell death of cholinergic neurons is the central hallmark of Alzheimer’s disease. Cholinergic neurons are located in distinct areas of the brain, and neurons located in the septum/diagonal band of Broca project to the hippocampus, while neurons located in the basal nucleus of Meynert innervate the whole cortex. In the striatum the cholinergic neurons are mainly large interneurons. Already in 1983, Keller et al. (1983) reported the presence of cholinergic cells and nerve fibers in organotypic cultures of the septum and hippocampus. This was further developed and characterized by Gähwiler et al. (1990), who showed for the first time that NGF is required to maintain cholinergic septal organotypic neurons. We ourselves focused on the cholinergic neurons of the nucleus basalis of Meynert and verified the important role of NGF for cholinergic neurons, thus supporting the view that organotypic brain slices may be a potent tool for studying neurodegeneration of cholinergic neurons linking to Alzheimer’s disease (Weis et al., 2001; Humpel and Weis, 2002).

NGF is an example of how *in vitro* experiments can revolutionize a whole scientific field (Levi-Montalcini et al., 1995). The trophic effect of NGF was first shown in spinal cord ganglia *in vitro* (Crain and Peterson, 1974; Sedel et al., 1999), and the first effects of NGF on cholinergic neurons also *in vitro* (Honegger and Lenoir, 1982). These important *in vitro* experiments have led to many *in vivo* works. It is well-established that NGF is the most potent neuroprotective molecule to support the survival of cholinergic neurons in organotypic brain slice cultures. In our hands cholinergic neurons of the nucleus basalis of Meynert survive well when incubated from the beginning with 10 ng/ml NGF, and we find approx. 100 neurons/slice (Weis et al., 2001). However, when slices are incubated without NGF, nearly 10 neurons/slice are found, but do not look healthy.

Finally, the organotypic slice model, especially the hippocampal formation, has served as a good model for studying beta-amyloid toxicity as a model for Alzheimer’s disease. Several groups have studied cytochemical changes (Suh et al., 2008; Frozza et al., 2009) and apoptotic cell death (Allen et al., 1995; Chong et al., 2006) after beta-amyloid toxicity, protective effects mediating oxidative stress (Bruce et al., 1996; Clapp-Lilly et al., 2001), the modulating effects of different pro-inflammatory stimuli (Harris-White et al., 1998), intracellular pathways (Nassif et al., 2007; Tardito et al., 2007) as well as tau phosphorylation (Johansson et al., 2006). Prasanthi et al. (2011) showed that endoplasmatic reticulum stress-mediated transcriptional activation in organotypic adult rabbit hippocampal slices triggered with 27-hydroxycholesterol. Schrag et al. (2008) found that neurons in organotypic slices from adult dwarf mice are resistant to beta-amyloid induced tau-hyperphosphorylation and changes in apoptosis-regulatory protein levels. Using rat cortical neurons in culture and entorhinal-hippocampal organotypic slices, Alberdi et al. (2010) found that beta-amyloid oligomers significantly induced intracellular Ca^2+^ and apoptotic cell death through a mechanism requiring NMDA and AMPA receptor activation. In organotypic hippocampal slice cultures it was shown (Kreutz et al., 2011) that ganglioside GM1 exhibited a neuroprotective activity on beta-amyloid-induced apoptosis. Finally, we showed for the first time that organotypic brain slices develop beta-amyloid “plaque-like deposits” when incubated for several weeks under low acidic pH with apolipoprotein E4 (Marksteiner and Humpel, 2008).

## DOPAMINERGIC NEURONAL CO-CULTURES AS A MODEL FOR PARKINSON’S DISEASE?

The major advantage of organotypic brain slices is that it permits cells from two or more functionally related brain areas to be cultured simultaneously. A first publication on co-cultures of organotypic tissue reported the innervation of fetal rodent skeletal muscle by spinal cord (Peterson and Crain, 1970). Many other co-cultures have been studied meanwhile, including septo-hippocampal, cortico-striatal, cortico-spinal, cortico-thalamic and entorhinal-hippocampal (Woodhams and Atkinson, 1996). The most studied co-culture system, however, is the striatonigral system, because it plays an important role in Parkinson’s disease. Such co-cultures allow the long-distance nerve fiber growth and connectivity between neuronal populations and brain areas to be studied and characterized.

Cell death of dopaminergic neurons is the central hallmark of Parkinson’s disease. Dopaminergic neurons are located in the ventral mesencephalon (vMes) and neurons of the substantia nigra project into the dorsal striatum (nigrostriatal pathway), while neurons of the ventral tegmental area project into the ventral striatum (meso-limbic pathway). Organotypic brain slices of the vMes and the striatum are well-established, and several exciting papers describe the nigrostriatal pathway in slices. Survival of dopaminergic neurons in the substantia nigra in organotypic brain slices was already reported in 1982 (Hendelman et al., 1982) and further characterized in 1989 (Jaeger et al., 1989). Pioneering work has been done by Zimmer’s groups, who detailed the survival and nerve fiber growth of dopaminergic nigrostriatal neurons (Ostergaard et al., 1990, 1991). We ourselves characterized mesencephalic dopamine neurons and observed that glial cell line-derived neurotrophic factor (GDNF) was essential for survival and nerve fiber growth (Schatz et al., 1999), which was verified by others (Jaumotte and Zigmond, 2005; af Bjerkén et al., 2007). This work provided the basis for further developing and characterizing the nigrostriatal nerve fiber innervation (Heine and Franke, 2014) and developing organotypic slices as an *in vitro* model for Parkinson’s disease (Stahl et al., 2009; Ullrich and Humpel, 2009b; Cavaliere et al., 2010; Daviaud et al., 2014).

The striatonigral tract is of special interest, because it degenerates in Parkinson’s disease. It has been reported that outgrowth of dopamine fibers from the mesencephalon occurs irrespective of the age of the donor rats, and a pronounced innervation of dopamine nerve fibers into the striatum has been seen (Ostergaard et al., 1990). The distance between the mesencephalon and the striatum was between 0.5 and 2.0 mm at the end of culturing. Thus, the dopaminergic fibers from the vMes could extend over a long distance, and it was reported that the maximum distance covered between striatonigral co-slices was 5.7 mm (Ostergaard et al., 1990). Franke et al. (2003) reported that in mesencephalic/striatal co-slices an extensive fiber bridge was observed in the co-cultures and that dopaminergic neurons develop their typical innervation pattern. Snyder-Keller et al. (2001) showed that the striatal patch/matrix organization was maintained in organotypic slice cultures taken from E19-P4 rats. We ourselves showed in a previous work that cultures of mesencephalic/striatal co-slices exhibit a large number of surviving dopamine neurons in the presence of GDNF and that intense fiber innervation is seen in striatal slices (Schatz et al., 1999; Zassler et al., 2003, 2005). Using sagittal brain slices we ourselves showed for the first time that dopamine neurons survive although the striatonigral pathway is not functional (Ullrich et al., 2011).

## THE VASCULAR SYSTEM IN ORGANOTYPIC SLICES

Brain capillaries constitute the BBB and innervate all areas of the brain. A first description of the vasculature of organotypic brain slices was given in 1975 (Wolff et al., 1975). Subsequently, Renkawek et al. (1976) characterized brain capillaries in organotypic cultures using relatively unselective butyryl cholinesterase stainings. We ourselves were one of the first to demonstrate at the cellular level that organotypic brain slices contain a strong network of laminin-positive brain capillaries (Moser et al., 2003, 2004). Laminin is a well-established basement membrane marker and excellently stains the vascular structures of the brain. We demonstrated that capillaries survive well in organotypic sections without any circulation (Moser et al., 2003). Although the capillaries are no longer functional and do not display any blood flow, it is likely that they express and secrete a cocktail of various molecules that may indeed also influence other cells in the slices including nerve fiber innervations (Moser et al., 2003; Kovács et al., 2011). Meanwhile brain vessels in organotypic cultures have been well studied, and especially the neurovascular unit and the interaction of endothelial cells with pericytes is coming under intense investigation (Camenzind et al., 2010; Chip et al., 2013, 2014; Morin-Brureau et al., 2013; Zehendner et al., 2013; Mishra et al., 2014).

The testing of pro- or anti-angiogenic growth factors is important when studying angiogenesis and revascularization in organotypic slices (Morin-Brureau et al., 2011). Especially two growth factors are of particular interest, when exploring the vascular network: vascular endothelial growth factor (VEGF) and fibroblast-growth factor-2 (FGF-2, bFGF). VEGF and its tyrosine kinase receptors (VEGFR-1, flt-1 and VEGFR-2, flk-1/KDR) are key mediators of angiogenesis. They are usually expressed during embryonic development but are downregulated in the adult. Kremer et al. (1997) investigated for the first time the time-dependent expression of VEGFR-2 in cerebral slice cultures and found that VEGF and hypoxia upregulated VEGFR-2 expression. This was verified by Rosenstein et al. (1998), who found significant angiogenic effects after VEGF application in a dose-responsive manner in fetal, newborn and adult rat cortical slices, which was abolished by a VEGF neutralizing antibody. After VEGF application, explants from adult donors had enlarged, dilated vessels that appeared to be an expansion of the existing network (Rosenstein et al., 1998). Further, they found that these slice culture vessels expressed both VEGF receptors (Rosenstein et al., 1998). Interestingly, the same group showed that VEGF had a neurotrophic effect in fetal organotypic cortex explants, and it was suggested that VEGF has neuroprotective activity independent of a vascular component (Rosenstein et al., 2003). The effect of FGF-2 on the vascular network was contradictory. While Rosenstein et al. (1998) found that all FGF-2 treated slice cultures exhibited substantially fewer vascular profiles, Bendfeldt et al. (2007) showed that FGF-2 maintained blood vessels and preserved the composition of tight junctions in neonatal mouse brain slices. However, while moderate FGF-2 concentrations (0.5–5 ng/ml) markedly increased the number of vessels, an excess of FGF-2 (50 ng/ml) reduced the vessel density. This again here clearly points to the need to perform dose- as well as time-dependent experiments in testing the effects of exogenous stimuli in brain slice cultures.

There is a clear need to develop fast and simple *in vitro* models for a high-throughput screening of pro-angiogenic factors or angiogenic inhibitors (Staton et al., 2004). So far the most useful angiogenic assays include *in vivo* Matrigel plug and sponge and corneal neovascularization, the chick chorioallantoic membrane and aortic arch assays, the *in vitro* cellular (proliferation, migration, tube formation) and organotypic (aortic ring) assays (Auerbach et al., 2003; Staton et al., 2009). Most pro- or anti-angiogenic drugs have been tested in co-cultures of endothelial cells and pericytes or smooth muscle cells forming a tubular network, but organotypic brain slice cultures have to our knowledge not yet extensively used for screening pharmacological drugs. We ourselves used this model to investigate whether brain vessels degenerate, sprout or can grow over a lesion site. Using e.g. laminin-counterstained brain slices, we overlay this vascular network on a 6 × 6 grid in Photoshop and quantify the vascular network by counting the crossings in the 6 × 6 grid (Moser et al., 2003). Indeed, using such a model we previously showed that in adult brain cultures of adult transgenic Alzheimer mice, substance P and calcium channel blockers induced angiogenesis (Daschil et al., 2015). Further, we demonstrated that brain vessels in different organotypic brain slices can grow back together when exogenously stimulated (Ullrich and Humpel, 2009a).

Another important innovative approach is to co-culture brain endothelial cells with organotypic brain slices and build up an *in vitro* BBB. Indeed, Duport et al. (1998) developed and characterized such a model 15 years ago when they overlaid organotypic brain slices on an endothelial monolayer growing on permeable membranes and concluded that this model possesses characteristics of a BBB in situ including tightness. However, this model seems to be very complex and tricky, and we ourselves have never succeeded in setting up such a complex *in vitro* model, nor are we aware of other groups using such a system. Indeed, the development of a simple BBB model using only brain capillary endothelial cells (BCECs) is a very tricky and complex model and must include tight junctions and a close layer of BCEC to guarantee an electrically tight junction resistance. Thus, although this was powerful pioneering work, much more work is needed before this “slice-BBB model” can serve for further pharmacological use.

## HOW COMPARABLE ARE BRAIN SLICES WITH THE *IN VIVO* SITUATION?

The main question arises: how close are *in vitro* models to the *in vivo* situation? This is a complex question because another question may also be asked, namely how close are *in vivo* murine models to the human situation? In my opinion, *in vitro* models can help us gain more mechanistic insights, which then must be proven *in vivo* in the animal model. Vice versa, results in animal models must be proven in postmortem human material and finally in human imaging and therapeutic/diagnostic approaches. Primary as well as organotypic models definitely have their advantages and disadvantages. Both *in vitro* models may demonstrate proof of principle, which must subsequently be proven *in vivo*. Regarding organotypic brain slice cultures, the complex three-dimensional architecture is partly maintained, while pathways are largely disconnected but could also be re-established. Thus, as compared to primary single-cell cultures, such an organotypic slice culture model is at least closest to an *in vivo* situation.

However, at this point another question arises: do we want to study development or a mature adult situation. It is well known that developing neurons have different characteristics (dependence on growth factor, receptor expression, protein expression …) than do mature adult neurons. Thus, it needs to be proven whether and, if so, when cultured neurons derived from postnatal donors develop a mature phenotype and display the same molecular and cellular pattern as a mature adult neuron. Regarding dissociated neurons, this question can be neglected because primary dissociated neurons cannot be cultured for several weeks. However, organotypic brain slice cultures can be cultured for long times, and the question arises whether slices derived from postnatal donors and cultured for more than two months can represent a mature adult situation. Clearly, much more work is needed to fully answer this question. On the other hand, a similarly critical question suggests itself, namely whether a one-year-old mouse compares with the mature adult situation of a 40-year-old human.

## OUTLOOK

Taken together, the organotypic slice cultures are a potent *in vitro* system for studying many of the brain’s cells. However, there are several challenging options for further improving this model. (1) There is a clear need to reconstruct axotomized neuronal pathways to establish functional pathways. It will be necessary to improve growth factor applications and to target the inputs into the target regions. Brain slices could become a potent means of studying nerve regeneration across longer distances, and it is important to test the bridging of various substances, such as e.g. tubes of polyglycolic acid-collagen (Kiyotani et al., 1996) or other biomaterials, including growth factor-releasing scaffold nanostructures (Breen et al., 2009). (2) The development of adult organotypic brain slices (including human tissue) is one of the primary goals, since most researchers want to study changes in disease models and correlate to an adult situation. Whether the postnatally derived slices represent only a developing model and in no way compare with a mature adult situation is still the subject of discussion. (3) To overcome this problem long-term cultures are necessary. However, maintaining cultures over several months is time-consuming and keeping them under sterile conditions is tricky. (4) There is a definite need to couple brain slices and a BBB; such a complex model will allow the entry of substances directly into the brain to be studied and may simulate an *in vivo* situation even better. (5) As a perspective, it would be highly attractive to couple the slices and the vascular system with a tube perfusion system; this would permit the simulation of blood flow and the continuous supply of needed substances. Such a model would also allow the release of neurotransmitters or cytokines from brain slices to be measured. (6) Brain slices may also serve as diagnostic tools; e.g. coupling slices and electrode arrays or biochips (Maher et al., 1999; Kristensen et al., 2001) may provide direct and fast information on a cell type; brain slices could be directly perfused with human body fluids, such as e.g. cerebrospinal fluids or plasma. (7) Brain slices could be coupled with stem cells to study neurogenesis, or neurogenesis could be stimulated by e.g. excitotoxic cell death chemicals (Daviaud et al., 2013; Mazzone et al., 2013). This could also be done to build up whole functional brain areas. Initial pioneering work establishing cerebral organoids was recently published by Lancaster et al. (2013). Moreover, exogenous cells modified, manipulated or genetically engineered could further improve the slice model. Choi et al. (2014) recently established a three-dimensional human neural cell culture model of Alzheimer’s disease.

In conclusion, organotypic slice cultures are an innovative and potent *in vitro* method that permits several cell types of the brain to be studied in a complex network. Slices can be cultured as single slices or as whole-brain sagittal slices. Further improvement and new techniques might make it possible to prepare whole functional brain models, possibly forming a complex artificial brain including a BBB. Such a complex brain culture system might provide an excellent model for studying neurodegenerative brain diseases, including e.g. Alzheimer’s and Parkinson’s disease. Finally, organotypic brain slice cultures markedly reduce the number of severe animal experiments contributing to the 3Rs (reduce, refine, replace).

## Abbreviations

BBB: blood–brain barrier
BCECs: brain capillary endothelial cells
FGF-2: fibroblast-growth factor-2
GDNF: glial cell line-derived neurotrophic factor
HEPES: 4-(2-hydroxyethyl)-1-piperazineethanesulfonic acid
NGF: nerve growth factor
NMDA: N-methyl-d-aspartate
PBS: phosphate-buffered saline
VEGF: vascular endothelial growth factor
vMes: ventral mesencephalon
